# Systemic immune-inflammation index is associated with white matter hyperintensity volume

**DOI:** 10.1038/s41598-022-11575-0

**Published:** 2022-05-05

**Authors:** Ki-Woong Nam, Hyung-Min Kwon, Han-Yeong Jeong, Jin-Ho Park, Hyuktae Kwon

**Affiliations:** 1 Departments of Neurology, Seoul National University College of Medicine, Seoul, South Korea; 2 Department of Neurology, Seoul National University College of Medicine and Seoul Metropolitan Government-Seoul National University Boramae Medical Center, 20 Boramae-ro 5-gil, Dongjak-gu, Seoul, 07061 South Korea; 3 Departments of Family Medicine, Seoul National University College of Medicine and Seoul National University Hospital, 101 Daehak-ro, Jongno-gu, Seoul, 03080 South Korea

**Keywords:** Neurology, Cerebrovascular disorders

## Abstract

Systemic immune-inflammation index (SII) is a novel inflammatory marker based on the composition ratio of blood cell counts. In this study, we evaluated the association between the SII and cerebral small vessel disease (cSVD) in health check-up participants. We evaluated participants from our health check-up registry between 2006 and 2013. The SII was calculated using the following formula: SII = (platelet count × neutrophil count)/lymphocyte count. cSVD was assessed by considering white matter hyperintensity (WMH) volume, lacunes, and cerebral microbleeds (CMBs). A total of 3187 participants were assessed. In multivariable linear regression analysis, the SII was significantly related to WMH volume [*β* = 0.120, 95% confidence interval (CI) 0.050–0.189]. However, lacunes and CMBs showed no statistical significance with the SII. In the subgroup analysis by age, the SII was significantly associated with WMH volume only in participants aged ≥ 60 years (*β* = 0.225, 95% CI 0.068–0.381). In conclusion, a high SII was associated with cSVD. Since this association was more pronounced in WMH than in lacunes or CMBs, WMH might be closer to the inflammation-related pathological mechanisms.

## Introduction

Cerebral small vessel diseases (cSVDs) are subclinical pathologies mainly observed in older adults and comprise various subtypes including white matter hyperintensity (WMH), lacunes of presumed vascular origin, and cerebral microbleeds (CMBs)]^1–3^. With the worldwide increase in population age, cSVD prevalence is gradually increasing^2,3^. Large cSVD lesions are associated with cognitive dysfunction, gait disturbance, and dysphagia. Furthermore, cSVD is clinically important because it may increase the risk of dementia and stroke^1,3–5^. The cSVD subtypes vary distinct in shape, but tend to cluster together on brain images^6^. Therefore, studies have been conducted for finding a common pathological mechanism penetrating these subtypes^1,2,4,7^.

One of these mechanisms is inflammation. Chronic systemic inflammation can affect various vascular walls from small arterioles to large arteries through varied mechanisms (including endothelial dysfunction, lipohyalinosis, and atherosclerosis), leading to the development of cSVD^4,8^. Several previous studies have already shown that cSVD is closely related to various inflammatory markers^9–11^. Furthermore, chronic inflammation is asymptomatic and affects the cerebrovascular environment slowly over a long period^12,13^. Therefore, classification of high-risk groups using appropriate inflammatory markers and individualized treatment are required^9^.

Recently, several effective inflammatory markers based on the ratio between various blood cell counts have been proposed, including the systemic immune-inflammation index (SII)^14,15^. The SII can easily be obtained using neutrophil, lymphocyte, and platelet counts^16^. To date, the SII has been closely associated with cancer, dementia, atherosclerosis, stroke, and cardiovascular diseases^17–20^. Therefore, it might be closely linked with cSVD as well; however, this aspect has not yet been investigated. Furthermore, the SII not only reflects systemic inflammation but also reflects the balance between innate and adaptive immunity, which may provide interesting insight into cSVD pathophysiology^19^.

With the development of brain imaging technology, cSVD lesions have been more often found incidentally during health check-ups. Here, we evaluated the association between the SII and cSVD in health check-up participants. Additionally, we aimed to confirm the relationship between the SII and each cSVD subtype and to determine if any pathology had a strong association with the SII.

## Results

A total of 3,187 participants were evaluated (mean age: 57 years, male sex: 53.9%). The maximum age of the participants was 86 years, and the minimum age was 30 years. The mean SII was 410.60 ± 240.84; median WMH volume, 1.10 [0.20–2.70] mL; and prevalence of lacunes and CMBs, 241 (7.6%) and 131 (4.1%), respectively. Detailed baseline characteristics are shown in the Supplementary Table 1.

**Figure 1:**
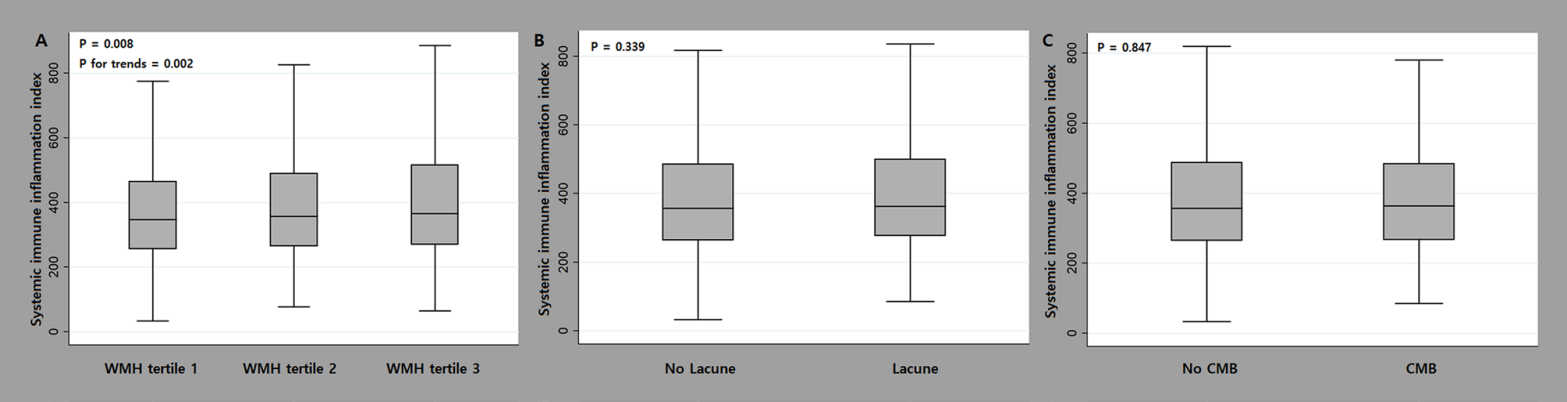
The relationship between the systemic immune-inflammation index and each subtype of cerebral small vessel disease. The systemic immune-inflammation index (SII) showed a significant association with white matter hyperintensity volume tertile (*P* = 0.008) in a positive dose–response manner (*P* for trend = 0.002). Conversely, the SII did not show any significant association with lacunes (*P* = 0.339) or cerebral microbleeds (*P* = 0.847).

In the univariate linear regression analysis, WMH volume was associated with age, hypertension, diabetes, ischemic heart disease, current smoking, SII, ICAS, and ECAS. In the multivariable linear regression analysis, the SII was significantly related to WMH volume after adjusting for confounders [*β* = 0.120, 95% confidence interval (CI) 0.050–0.189]. Age (*β* = 0.051, 95% CI 0.047–0.055), hypertension (*β* = 0181, 95% CI 0.097–0.266), and diabetes (*β* = 0.144, 95% CI 0.042–0.247) were associated with WMH volume but independent of the SII (Table 1). However, lacunes and CMBs were not significantly related to the SII (shown in Fig. 1 and Supplementary Tables 2 and 3).

**Table 1:** Simple and multiple linear regression analyses between possible predictors and the square root of white matter hyperintensity volume (these variables were transformed into square root scales).

	Univariate analysis		Multivariate analysis	
	*β* (95% CI)	*P*-value	*β* (95% CI)	*P*-value
Age	0.054 (0.050 to 0.058)	< 0.001	0.051 (0.047 to 0.055)	< 0.001
Male sex	0.001 (− 0.076 to 0.078)	0.979	0.032 (− 0.040 to 0.105)	0.381
Body mass index	0.006 (− 0.007 to 0.018)	0.352	…	…
Hypertension	0.454 (0.364 to 0.545)	< 0.001	0.181 (0.097 to 0.266)	< 0.001
Diabetes	0.441 (0.331 to 0.551)	< 0.001	0.144 (0.042 to 0.247)	0.006
Hyperlipidemia	0.039 (− 0.050 to 0.127)	0.391	…	…
Ischemic heart disease	0.206 (0.006 to 0.406)	0.043	− 0.085 (− 0.264 to 0.094)	0.351
Current smoking	− 0.238 (− 0.343 to − 0.132)	< 0.001	− 0.003 (− 0.104 to 0.098)	0.955
WBC counts	0.049 (0.026 to 0.072)	< 0.001	…	…
Neutrophil counts	0.079 (0.050 to 0.109)	< 0.001	…	…
Lymphocyte counts	− 0.030 (− 0.099 to 0.039)	0.394	…	…
Platelet counts	− 0.001 (− 0.001 to 0.000)	0.051	…	…
SII^a^	0.160 (0.083 to 0.238)	< 0.001	0.120 (0.050 to 0.189)	0.001
Hs-CRP^a^	0.017 (− 0.008 to 0.043)	0.182	…	…
ICAS	0.524 (0.301 to 0.748)	< 0.001	0.157 (− 0.044 to 0.358)	0.126
ECAS	0.856 (0.499 to 1.213)	< 0.001	0.318 (− 0.002 to 0.638)	0.052

*WBC* white blood cell, *SII* systemic immune-inflammation index, *hs-CRP* high-sensitivity C-reactive protein, *ICAS* intracranial atherosclerosis, *ECAS* extracranial atherosclerosis.

^a^These variables were transformed into log scales.

In the subgroup analysis by age, participants aged ≥ 60 years had greater WMH volume (2.40 [0.93–5.00] mL versus 0.65 [0.06–1.69] mL, *P* < 0.001) and higher SII (367.44 [271.39–514.84] versus 350.90 [261.46–474.53], *P* = 0.005) than participants aged < 60 years. The SII was significantly associated with WMH volume only in participants aged ≥ 60 years (*β* = 0.225, 95% CI 0.068–0.381). No statistical significance was found in participants younger than 60 years (Table 2). In the subgroup analysis by sex, the SII showed a significant association with WMH volume in both males (*β* = 0.127, 95% CI 0.034–0.221) and females (*β* = 0.114, 95% CI 0.010–0.218), with a stronger tendency in males (Table 3).

**Table 2:** Multivariable linear regression analysis of possible predictors for white matter hyperintensity volume (these variables were transformed into square root scales) according to the age.

	Age < 60 years (n = 2036)		Age ≥ 60 years (n = 1151)	
	*β* (95% CI)	*P*-value	*β* (95% CI)	*P*-value
Male sex	0.032 (− 0.042 to 0.106)	0.396	− 0.030 (− 0.188 to 0.127)	0.704
Hypertension	0.162 (0.069 to 0.255)	0.001	0.311 (0.146 to 0.476)	< 0.001
Diabetes	0.308 (0.192 to 0.424)	< 0.001	0.143 (− 0.049 to 0.336)	0.144
Ischemic heart disease	− 0.085 (− 0.301 to 0.131)	0.442	− 0.013 (− 0.336 to 0.310)	0.937
Current smoking	− 0.100 (− 0.193 to − 0.008)	0.033	− 0.164 (− 0.441 to 0.113)	0.245
SII^a^	0.021 (− 0.048 to 0.091)	0.550	0.225 (0.068 to 0.381)	0.005
ICAS	0.211 (− 0.033 to 0.455)	0.090	0.261 (− 0.101 to 0.622)	0.158
ECAS	0.124 (− 0.365 to 0.614)	0.619	0.573 (0.067 to 1.078)	0.026

*SII* systemic immune-inflammation index, *ICAS* intracranial atherosclerosis, *ECAS* extracranial atherosclerosis.

^a^These variables were transformed into log scales.

**Table 3:** Multivariable linear regression analysis of possible predictors for white matter hyperintensity volume (these variables were transformed into square root scales) according to the sexual difference.

	Male (n = 1718)		Female (n = 1469)	
	*β* (95% CI)	*P*-value	*β* (95% CI)	*P*-value
Age	0.050 (0.044 to 0.055)	< 0.001	0.052 (0.046 to 0.058)	< 0.001
Hypertension	0.159 (0.050 to 0.267)	0.004	0.203 (0.068 to 0.338)	0.003
Diabetes	0.122 (-0.001 to 0.246)	0.053	0.191 (0.012 to 0.370)	0.037
Ischemic heart disease	− 0.085 (− 0.314 to 0.145)	0.470	− 0.096 (− 0.381 to 0.188)	0.507
Current smoking	− 0.032 (− 0.139 to 0.076)	0.562	0.158 (− 0.135 to 0.450)	0.291
SII^a^	0.127 (0.034 to 0.221)	0.008	0.114 (0.010 to 0.218)	0.032
ICAS	0.135 (− 0.139 to 0.408)	0.334	0.177 (− 0.122 to 0.475)	0.246
ECAS	0.517 (0.126 to 0.908)	0.010	− 0.023 (− 0.573 to 0.528)	0.936

*SII* systemic immune-inflammation index, *ICAS* intracranial atherosclerosis, *ECAS* extracranial atherosclerosis.

^a^These variables were transformed into log scales.

In our data, the SII showed a positive association with hypertension, diabetes, current smoking, high-sensitivity C-reactive protein (hs-CRP), WMH volume, ICAS, and ECAS. Whereas, it was negatively correlated with body mass index (Supplementary Table 4).

## Discussion

Here, high SII was associated with WMH volume in health check-up participants. The SII did not show any association with lacunes or CMBs. Thus, inflammation-related pathology may be more closely related to WMH than other cSVD subtypes.

The exact mechanisms explaining the close association between the SII and WMH volume are unclear. However, considering the role of the SII as a chronic inflammatory marker, we suggest several plausible hypotheses. First, endothelial dysfunction should be considered^21^. Normal endothelial cells secrete vasodilators (e.g., nitric oxide, prostacyclin) and antithrombotic agents^14,19,20^. In chronic inflammation conditions, activated neutrophils and platelets interfere with vasodilators secretion, ultimately leading to blood–brain barrier (BBB) disruption^14,22^. BBB disruption then leads to the release of various toxic metabolites into the periventricular spaces, damaging the surrounding neural tissue^23,24^. Additionally, the clearance of interstitial fluid through the glymphatic pathway is also disturbed^14^. These phenomena induce pathological changes in white matter areas, resulting in WMH. Second, chronic diffuse hypoperfusion may be involved. Inflammation is closely related to atherosclerosis in the large vessels^25^, as confirmed by studies investigating the association between the SII and coronary or carotid artery stenosis^19,20^. ICAS and ECAS resulting from this process can induce diffuse hypoperfusion of the brain. The last hypothesis is the presence of numerous vascular risk factors in participants with high SII. People with chronic inflammation tend to develop several metabolic or cardiovascular diseases, as demonstrated in our data. Most of these risk factors are closely related to WMH development.

Additionally, the SII can be used as an indicator of immune balance, along with its role as a simple inflammatory marker^26^. Therefore, considering the characteristics of each component of the SII formula, several interpretations can also be made for the close relationship between the SII and WMH volume. First, a high SII may indicate enhanced innate immunity and attenuated adaptive immunity^19,26^. Neutrophils, the core of innate immunity, damage neural tissue integrity by secreting various cytokines, chemokines, metalloproteinases, elastases, and proteolytic enzymes^14,20,26,27^. Platelet is also one of the components constituting innate immunity, and activated platelets can also contribute to neuronal cell death to some extent^20,27,28^. In adaptive immunity, lymphocytes promote healing of damaged tissues through the secretion of substances such as interleukin-10^14,28^. Therefore, individuals with a high SII, the neural tissue will be extensively damaged and not recover properly even from the subtle ischemic insults. Second, lymphocyte numbers may decrease under the influence of stress hormone secretion (e.g., cortisol) in many chronic stressful situations^20,27^. Thus, a low lymphocyte count may indicate a high burden of underlying disease, including the aforementioned vascular risk factors, and these diseases may in turn exacerbate WMH. In future studies, if we analyze broadly including other cell lines (e.g., monocytes, NK cells, eosinophils, basophils, B/T cells) and transmitters, the role of innate and adaptive immunity in WMH pathophysiology may be more clearly defined.

Interestingly, the close association between the SII and WMH volume in our study was significant only in older participants aged ≥ 60 years. This might be related to the aging-related changes in the homeostatic maintenance of our body’s inflammation and immunity (e.g., inflammaging, immunosenescence, and homeostenosis)^22,24,29^. Also, age is the strongest risk factor for WMH volume. Thus, it is also necessary to consider the possibility that the act of dividing subgroups based on age paradoxically showed statistical significance only in one subgroup. In the comparison by sex, the SII showed a stronger association in males than in females. However, this difference could not be confirmed because there was only a difference in the degree and both sexes showed statistical significances.

There are several limitations to interpreting our results. First, this was a retrospective cross-sectional study, which only allowed interpretation of associations. Therefore, further prospective studies are needed to obtain causal relationships. Second, we only saw the correlation between the SII measured at a single timepoint and the WMH volume. WMH progresses slowly and chronically. As such, we cannot determine when this radiological parameter arose. If we measured the SII of several timepoints over a long period of time and analyzed the relationship with the change in WMH volume, we would have been able to determine the causal relationship or the sequence of pathophysiological events according to the time change. Third, since the size of the human brain can vary according to age and sex, more accurate results may be obtained if the analysis was performed using the standardized WMH volume corrected for the whole brain size. Fourth, since SII is measured from peripheral blood, it is not a direct indicator reflecting inflammation of the central nervous system. Although previous studies have demonstrated associations of various systemic inflammatory markers with central nervous system inflammation or neuroinflammatory diseases, cation is still needed in the interpretation of our results. Last, due to technical limitations, we could not measure periventricular WMH and subcortical WMH separately. If the correlation with SII could be examined by measuring the lesions in the two areas, it would have been great help in elucidating the pathophysiology of WMH.

In conclusion, we demonstrated that the SII was associated with WMH volume in health check-up participants. Moreover, the SII is relatively inexpensive, fast, and convenient because it can be obtained with a simple blood test. Therefore, we believe that SII has potential as a screening test for classifying high-risk groups according to WMH for individualized treatment. However, future studies should verify this possibility.

## Methods

### Study population

We retrospectively assessed the medical records of participants consecutively enrolled in the health check-up registry at the Seoul National University Hospital Health Promotion Center between January 2006 and December 2013. Our center conducts extensive evaluations as part of the routine health check-up, including brain magnetic resonance imaging (MRI), magnetic resonance angiography (MRA), and laboratory examinations. Among them, participants who met the following exclusion criteria were excluded: (1) history of stroke or severe neurological disease, (2) age < 30 years, or (3) no blood cell count data. Additionally, participants with severe systemic inflammatory conditions including hemato-oncologic disease, use of immunosuppressants, severe hepatic or renal disease, major surgery or severe trauma, or active infection within the previous 2 weeks were excluded^14^. Finally, a total of 3187 participants were analyzed.

The Institutional Review Board (IRB) of the Seoul National University Hospital approved the study (number: 1502-026-647). The requirement for informed consent from participants was waived by the IRB because of the retrospective design and use of de-identified information. All experiments were performed in accordance with the Declaration of Helsinki and relevant guidelines and regulations.

### Clinical assessments

The demographic, clinical, and laboratory factors assessed included age, sex, body mass index, hypertension (use of anti-hypertensive drugs, ≥ 140 mmHg systolic blood pressure, or ≥ 90 mmHg diastolic blood pressure), diabetes (use of glucose-lowering drugs, or ≥ 6.5% hemoglobin A1c levels), hyperlipidemia (use of lipid-lowering drugs, ≥ 240 mg/dL total cholesterol levels, or ≥ 160 mg/dL low-density lipoprotein cholesterol levels), ischemic heart disease, and current smoking.

After 12 h of overnight fasting, laboratory examinations were performed including inflammatory markers [e.g., white blood cell (WBC) counts, hs-CRP]. Blood cell samples were collected in calcium ethylenediaminetetraacetic acid tubes, and differential blood count was obtained using an auto-analyzer at our center^14^. The SII was calculated using the following formula: SII = (platelet count × neutrophil count)/lymphocyte count^16^.

### Radiological assessments

The Participants underwent brain MRI and MRA using 1.5-T MR scanners (Signa, GE Healthcare, Milwaukee, WI, or Magnetom SONATA, Siemens, Munich, Germany). As part of the health check-ups, the participants’ medical history taking, blood test, and brain imaging were performed on the same day. The detailed MRI acquisition parameters were as follows: basic slice thickness = 5 mm, T1-weighted images [repetition time (TR)/echo time (TE) = 500/11 ms], T2-weighted images (TR/TE = 5000/127 ms), T2 fluid-attenuated inversion recovery images (TR/TE = 8800/127 ms), T2-gradient echo images (TR/TE = 57/20 ms), and three-dimensional time-of-flight MRA images (TR/TE = 24/3.5 ms, slice thickness = 1.2 mm).

cSVD was assessed by considering WMH volume, lacunes of presumed vascular origin (= lacunes), and CMBs as the main outcome variables. WMH volume was quantitatively measured using Medical Imaging Processing, Analysis, and Visualization software (MIPAV, version, 11.0.0, National Institutes of Health, Bethesda, MD, USA) as in previous studies^14^. For volume measurements, we obtained imaging data from converted DICOM files. Then, using the difference in shading, we were able to designate the borderline of the WMH lesion as semi-automated. The volume of WMH was automatically calculated through the sum of the areas secured in each slide. Lacunes were defined as asymptomatic, well-defined lesions 3–15 mm in size, with signal characteristics such as cerebrospinal fluid on T1- or T2-weighted images^1^. CMBs were defined as focal round lesions < 10 mm in size with low signal characteristics on T2-gradient echo images^1^. Intracranial atherosclerosis (ICAS) and extracranial atherosclerosis (ECAS) were defined as occlusion or more than 50% stenosis of the intracranial and extracranial vessels in time-of-flight MRA images^30,31^. Radiological parameters were rated by two neurologists (K.-W.N. and H.-Y.J.), and disagreements were resolved by discussion with a third rater (H.-M.K.).

### Statistical analysis

Univariate analysis was conducted to identify the possible predictors of WMH volume using simple linear regression analysis. Continuous variables with skewed data were transformed to log scales, whereas WMH volume was transformed to square root scale owing to many “zero” data. During univariate analysis, variables with *P* < 0.10 were included in the multivariable linear regression analysis along with age and sex. WBC, neutrophil, lymphocyte, and platelet counts, which were variables in the SII formula, were not included in the multivariable analysis^16^. Since lacunes and CMBs were binary outcomes, they were analyzed in the same way using logistic regression analysis.

To confirm the variation of inflammation effects because of age and sex, we performed a subgroup analysis stratified by age and sex. Additionally, since the SII is a marker that may be unfamiliar to neurologists, we analyzed the relationship between the SII and several demographic, clinical, and laboratory parameters via simple linear regression analysis to demonstrate the characteristics of patients with high SII. All statistical analyses were performed using SPSS version 20.0 (IBM Corp., Armonk, NY, USA), and statistical significance was set at *P* < 0.05.

## Supplementary Information

Supplementary Tables.
