# Supplementary material for: Systemic immune-inflammation index is associated with white matter hyperintensity volume
Source: Sci Rep. 2022 May 5;12:7379. doi: 10.1038/s41598-022-11575-0 (PMC9072679; doi:10.1038/s41598-022-11575-0)
Supplement: Supplementary file 1 — Supplementary Tables. [file 41598_2022_11575_MOESM1_ESM.pdf]

Supplemental Materials

Supplementary Table 1. Baseline characteristics of the study population (n = 3,187)

Age, years [IQR]	56 [50-63]
Male sex, n (%)	1,718 (53.9)
Body mass index, kg/m^2 [IQR]	24.05 [22.13-25.96]
Hypertension, n (%)	717 (22.5)
Diabetes, n (%)	439 (13.8)
Hyperlipidemia, n (%)	806 (25.3)
Ischemic heart disease, n (%)	122 (3.8)
Current smoking, n (%)	497 (15.6)
White blood cell counts, $\times 10^3$/ μL [IQR]	5.31 [4.40-6.37]
Neutrophil counts, $\times 10^3$/ μL [IQR]	2.87 [2.21-3.68]
Lymphocyte counts, $\times 10^3$/ μL [IQR]	1.86 [1.54-2.21]
Platelet counts, $\times 10^3$/ μL [IQR]	233 [201-269]
Systemic immune-inflammation index, $\times 10^3$/ μL [IQR]	357.10 [265.23-487.50]
High sensitivity C-reactive protein, mg/dL [IQR]	0.04 [0.01-0.15]
White matter hyperintensity volume, mL [IQR]	1.10 [0.20-2.70]
Lacune of presumed vascular disease, n (%)	241 (7.6)
Cerebral microbleeds, n (%)	131 (4.1)
Intracranial atherosclerosis, n (%)	96 (3.0)
Extracranial atherosclerosis, n (%)	37 (1.2)

Supplementary Table 2. Univariate and multivariate logistic regression analyses between possible predictors and lacunes

	Univariate		Multivariate	
	**Crude OR (95% CI)**	***P*-value**	**Adjusted OR (95% CI)**	***P*-value**
Age	1.09 [1.07-1.11]	< 0.001	1.08 [1.07-1.10]	< 0.001
Male sex	1.06 [0.81-1.38]	0.678	1.06 [0.81-1.40]	0.659
Body mass index	1.02 [0.97-1.06]	0.446	…	…
Hypertension	2.04 [1.54-2.69]	< 0.001	1.40 [1.04-1.88]	0.026
Diabetes	2.08 [1.52-2.85]	< 0.001	1.36 [0.97-1.91]	0.072
Hyperlipidemia	1.10 [0.82-1.48]	0.542	…	…
Ischemic heart disease	1.48 [0.82-2.68]	0.190	…	…
Current smoking	0.79 [0.53-1.16]	0.225	…	…
WBC counts	1.07 [1.00-1.16]	0.065	…	…
Neutrophil counts	1.02 [1.00-1.03]	0.044	…	…
Lymphocyte counts	0.98 [0.96-0.99]	0.007	…	…
Platelet counts	1.00 [1.00-1.00]	0.046	…	…
SII*	1.14 [0.87-1.49]	0.333	0.94 [0.71-1.26]	0.693
Hs-CRP*	1.11 [1.02-1.21]	0.015	1.06 [0.96-1.16]	0.252
ICAS	2.35 [1.33-4.14]	0.003	1.41 [0.78-2.57]	0.258
ECAS	1.08 [0.33-3.54]	0.899	…	…

WBC = white blood cell, SII = systemic immune-inflammation index, hs-CRP = high-sensitivity C-reactive protein, ICAS = intracranial atherosclerosis, ECAS = extracranial atherosclerosis

*These variables were transformed into log scales.

Supplementary Table 3. Univariate and multivariate logistic regression analyses between possible predictors and cerebral microbleeds

	Univariate		Multivariate	
	Crude OR (95% CI)	*P*-value	Adjusted OR (95% CI)	*P*-value
Age	1.00 [0.98-1.02]	0.794	1.00 [0.98-1.02]	0.750
Male sex	0.65 [0.46-0.92]	0.015	0.67 [0.47-0.96]	0.029
Body mass index	0.95 [0.89-1.00]	0.065	0.96 [0.90-1.01]	0.135
Hypertension	1.22 [0.82-1.82]	0.334	…	…
Diabetes	1.13 [0.70-1.85]	0.613	…	…
Hyperlipidemia	1.04 [0.69-1.54]	0.867	…	…
Ischemic heart disease	1.00 [0.40-2.48]	0.995	…	…
Current smoking	1.10 [0.69-1.75]	0.699	…	…
WBC counts	0.94 [0.84-1.05]	0.254	…	…
Neutrophil counts	1.00 [0.98-1.02]	0.935	…	…
Lymphocyte counts	1.00 [0.98-1.02]	0.864	…	…
Platelet counts	1.00 [1.00-1.00]	0.771	…	…
SII*	0.98 [0.69-1.40]	0.925	0.96 [0.68-1.37]	0.841
Hs-CRP*	0.96 [0.85-1.08]	0.485	…	…
ICAS	1.02 [0.37-2.81]	0.978	…	…
ECAS	1.34 [0.32-5.63]	0.691	…	…

WBC = white blood cell, SII = systemic immune-inflammation index, hs-CRP = high-sensitivity C-reactive protein, ICAS = intracranial atherosclerosis, ECAS = extracranial atherosclerosis

*These variables were transformed into log scales.

Supplementary Table 4. Univariate linear regression analysis between systemic immune-inflammation index and risk factors

	Univariate analysis	
	β (95% CI)	*P*-value
Age	0.001 (-0.001 to 0.003)	0.193
Male sex	-0.022 (-0.057 to 0.012)	0.200
Body mass index	-0.006 (-0.012 to -0.001)	0.031
Hypertension	0.059 (0.018 to 0.100)	0.005
Diabetes	0.075 (0.026 to 0.125)	0.003
Hyperlipidemia	0.039 (-0.001 to 0.078)	0.055
Ischemic heart disease	-0.058 (-0.148 to 0.031)	0.201
Current smoking	0.051 (0.004 to 0.098)	0.035
High sensitivity C-reactive protein*	0.081 (0.070 to 0.092)	< 0.001
White matter hyperintensity volume†	0.032 (0.017 to 0.047)	< 0.001
Lacune of presumed vascular disease	0.032 (-0.033 to 0.097)	0.333
Cerebral microbleeds	-0.004 (-0.090 to 0.082)	0.925
Intracranial atherosclerosis	0.128 (0.028 to 0.228)	0.012
Extracranial atherosclerosis	0.233 (0.073 to 0.393)	0.004

*These variables were transformed into log scales.

†These variables were transformed into square root scales.
